# Dimethyl Fumarate Ameliorates Paclitaxel-Induced Neuropathic Pain in Rats

**DOI:** 10.7759/cureus.28818

**Published:** 2022-09-06

**Authors:** Jagjit Singh, Surabhi Thapliyal, Ashish Kumar, Pranoy Paul, Nitesh Kumar, Manisha Bisht, Manisha Naithani, Shalinee Rao, Shailendra S Handu

**Affiliations:** 1 Department of Pharmacology, All India Institute of Medical Sciences, Rishikesh, Rishikesh, IND; 2 Department of Biochemistry, All India Institute of Medical Sciences, Rishikesh, Rishikesh, IND; 3 Department of Pathology, All India Institute of Medical Sciences, Rishikesh, Rishikesh, IND

**Keywords:** peripheral neuropathy, nrf2, mapk, dimethyl fumarate, cipn, central sensitization, antioxidant

## Abstract

Background

Paclitaxel (PTX)-induced peripheral neuropathy (PIPN) is nonresponsive to the currently available analgesics. Previous studies have shown the role of oxidative stress and central sensitization in the development of peripheral neuropathy. Dimethyl fumarate (DMF) acts as a nuclear factor erythroid-2-related factor 2 (Nrf2) activator with neuroprotective benefits and is approved for use in multiple sclerosis.

Materials and methods

In the current research, we evaluated the efficacy of DMF on paclitaxel-induced peripheral neuropathy in rats. Every alternate day for one week, paclitaxel 2 mg/kg dose was injected to establish a rat model of PIPN. Animals were treated with 25 mg/kg and 50 mg/kg of DMF. All the animals were assessed for thermal hyperalgesia, cold allodynia, and mechanical allodynia once a week. The gene expression of Nrf2 and the levels of pro-inflammatory mediators (interleukin (IL)-6, tumor necrosis factor-alpha (TNF-α), and IL-1β) were quantified in the sciatic nerves of these rats. The levels of p38 mitogen-activated protein kinase (MAPK) and brain-derived neurotrophic factor (BDNF) were quantified in the dorsal horn of the spinal cord.

Results

DMF significantly attenuated paclitaxel-induced thermal hyperalgesia and cold/mechanical allodynia. A significant decrease in the levels of pro-inflammatory cytokines with the levels of p38 MAPK and BDNF was observed in the DMF-treated animals. DMF treatment significantly upregulated the gene expression of Nrf2 in the sciatic nerve.

Conclusion

These findings suggest that DMF prevented the development of PIPN in rats through the activation of Nrf2 and the inhibition of p38 MAPK and BDNF.

## Introduction

Paclitaxel (PTX) is a plant alkaloid used as a chemotherapeutic agent in the treatment of breast, lung, and ovarian cancer. Unfortunately, paclitaxel causes a dose-limiting adverse effect termed peripheral neuropathy with the symptoms of pain, tingling, numbness, and sensitivity to cold in the stocking-glove areas [1]. Around 30%-40% of patients undergoing antineoplastic treatment experience these symptoms, which may persist for about 2-3 years [2,3]. Paclitaxel-induced peripheral neuropathy (PIPN) not only decreases the quality of life but also sometimes results in the cessation of chemotherapy when symptoms become extreme. Currently, available treatment provides minimal symptomatic relief and no or very little protection to peripheral nerves [4]. There have been multiple clinical and nonclinical studies, but none have come up with a breakthrough treatment for PIPN.

Evidence from various studies sheds light on oxidative stress as a particular component that contributes to the pathogenesis of peripheral neuropathy [5,6]. Several phytochemicals and drugs with reactive oxygen species (ROS)-scavenging properties have shown anti-neuropathic effects in multiple in vivo chemotherapy-induced peripheral neuropathy (CIPN) models [7]. In addition, some recent studies have shown the role of central sensitization in worsening neuropathic pain [8]. Nuclear factor erythroid-2-related factor 2 (Nrf2) acts as an essential modulator of in-house antioxidant defense [9]. It is a leucine zipper transcription factor that senses the change in redox activities and communicates with Kelch-like ECH-associated protein 1 inhibitory binding domain (KEAP 1) for antioxidant and anti-inflammatory responses. Nrf2 activation promotes a series of detoxification proteins through the antioxidant response element (ARE) [10]. Nrf2-ARE signaling cascade controls the production of multiple cytoprotective proteins, such as nucleotide adenosine diphosphate hydrogenase (NADPH), quinone oxidoreductase-1 (NQO1), glutathione (GSH) reduced, superoxide dismutase, and heme oxygenase-1 (HO-1) [11]. Furthermore, central sensitization leads to hypersensitivity to touch, heat, and cold. Spinal brain-derived neurotropic factor (BDNF) is one of the biomarkers for the central sensitization of pain. Previous studies show that the release of BDNF increases from microglial cells when there is peripheral nerve injury, which is mediated by adenosine triphosphate (ATP)-gated ionotropic purinoceptor, 2X4 (P2X4R) overexpression. This increase in expression parallels the increase in pain hypersensitivity [12]. This upregulation in P2X4Rs causes an increased influx of Ca+2, the activation of p38 mitogen-activated protein kinases (MAPKs), and a subsequent increase in the production and exocytosis of BDNF from microglia [13].

Dimethyl fumarate (DMF) has already been approved for multiple sclerosis. It acts by targeting Nrf2, increasing the transcription of antioxidant genes such as HO-1, GSH, and NADPH [14,15]. Studies in Nrf2-knockout mice revealed that Nrf2 is required to disseminate the therapeutic potential of fumarates [16]. An inhibitory effect of DMF has also been found on p38 MAPK, which is a crucial step in producing and releasing BDNF in the dorsal horn of the spinal cord.

Additionally, the activation of p38 MAPK in neuroinflammation leads to the release of pro-inflammatory cytokines such as interleukin (IL)-1β, IL-6, and tumor necrosis factor-alpha (TNF-α). This pathway further leads to the inactivation of Nrf2 and leads to a decrease in antioxidant gene transcription. Therefore, mitogen-activated protein kinase (MAPK) inhibition results in reduced inflammation. It stimulates antioxidant mechanisms by activating Nrf2 and reducing central sensitization [14,17]. This study was conducted to understand the effect of DMF in paclitaxel (PTX)-induced peripheral neuropathy through the activation of Nrf2 and the inhibition of p38 MAPK and BDNF in rats.

## Materials and methods

Animals

Thirty male Wistar rats weighing 200-250 g were used in this study. The animals were kept in a 12-hour light/dark cycle with a temperature of 23°C ± 2°C and relative humidity of 65%. Rats had ad libitum access to a standard pellet chow diet and tap water. The experiments were carried out according to the Committee for the Purpose of Control and Supervision of Experiments on Animals (CPCSEA) guidelines for using animals in research. The experimental procedures were approved by the Institutional Animal Ethics Committee (#IAEC/AIIMS/RISH/PHAR/01/19). A minimum possible number of rats was utilized in the experiment.

Study design

The animals were divided into five groups, and different drugs were given in all the groups as in Table 1.

**Table 1:** Different groups of animals and their respective treatment (n = 6) PTX: paclitaxel; GBP: gabapentin; DMF: dimethyl fumarate; CMC: carboxymethyl cellulose

Experimental group	Drugs and vehicle
Vehicle control	CMC 0.5%
PTX	Paclitaxel 2 mg/kg + CMC 0.5%
PTX + GBP	Paclitaxel 2 mg/kg + gabapentin 70 mg/kg
PTX + DMF 25	Paclitaxel 2 mg/kg + dimethyl fumarate 25 mg/kg
PTX + DMF 50	Paclitaxel 2 mg/kg + dimethyl fumarate 50 mg/kg

Drugs and chemicals

Paclitaxel (Sisco Research Laboratories (SRL) Pvt. Ltd., India) dissolved in CMC 0.5% was administered every alternate day for one week. Dimethyl fumarate (Sigma Aldrich, USA) dissolved in 0.5% carboxymethyl cellulose (CMC) was given orally (p.o.) for one week. Likewise, gabapentin (75 mg/kg) dissolved in 0.9% saline was given orally (p.o.) for one week. The treatment of gabapentin and dimethyl fumarate was given daily from day 0 to day 6. Enzyme-linked immunosorbent assay (ELISA) kits for interleukin (IL)-1β, IL-6, and tumor necrosis factor-alpha (TNF-α) were purchased from Invitrogen, USA. ELISA kit for the estimation of p38 MAPK was purchased from G Biosciences, USA.

Induction of peripheral neuropathy

Peripheral neuropathy was induced in animals by administering intraperitoneal (i.p.) injection of paclitaxel (PTX) 2 mg/kg on days 0, 2, 4, and 6. The assessment of pain threshold was done before administering PTX on day 0 and after administering PTX on days 7, 14, 21, and 28.

Assessment of thermal hyperalgesia

The instrument used in our study was Eddy’s Hot Plate Analgesio Meter (INCO Scientific, India). It consists of an electrically heated flat platform from which the animal cannot escape by itself. In this experiment, the hot plate was kept at 52°C-55°C. The individual rats were placed on the hot plate, and minimum threshold time was considered as soon as responses such as jumping or licking of the paws were seen. The maximum interval to last on the hot plate was 20 seconds to avoid any tissue injury. Eddy’s hot plate test was performed on days 0, 7, 14, 21, and 28 [18].

Assessment of mechanical allodynia

Before administering PTX, baseline readings for mechanical sensitivity were measured on day 0, and subsequent measurements were taken on days 7, 14, 21, and 28. The mechanical paw withdrawal threshold was evaluated using the von Frey aesthesiometer (Ugo Basile, Italy) on the left hind paw of the rats [19,20]. The minimum pressure that triggers the paw withdrawal was considered the mechanical withdrawal threshold. A series of stimulants with different weights were applied thrice with a five-minute interval in each measurement. The final reading was the mean of three observations for each rat [20].

Assessment of cold allodynia

The tail immersion test was used to assess cold allodynia. A beaker filled with ice-cold water was used to assess cold allodynia in rats. The water temperature was kept at 4°C, and the tip of the rat tail was dipped into that water. The latency to flick the tail was recorded. The maximum cutoff was kept at 20 seconds to avoid any tissue damage [21].

Enzyme-linked immunosorbent assay (ELISA)

The sciatic nerve and dorsal horns of spinal cord tissues were collected on day 28 and stored at -80°C. Specific kits were used to record the concentration of p38 MAPK (G Biosciences, USA) and BDNF (Invitrogen, USA) in the spinal cord dorsal horn. For pro-inflammatory cytokines (IL-6, IL-1β, and TNF-α), specific kits (Invitrogen, USA) were used to measure the concentration of each in the sciatic nerve. Experiments were conducted as per the manufacturers’ instructions in the respective kits.

Preparation of sciatic nerve tissues and staining techniques

Sciatic nerves were collected by sacrificing animals with a high dose of ketamine and xylazine solutions. A small portion of the sciatic nerve (1 cm) was isolated for histopathological examination. Samples were fixed in a 10% buffered formaldehyde solution. An automated tissue processor (Leica Biosystems, Nußloch, Germany) was used to process the tissues fixed in formaldehyde. Each sample was embedded in the paraffin wax block. Tissue samples in these blocks were cut into 5-6 µm each section. These sections were stained with hematoxylin and eosin (H&E), and Luxol fast blue (LFB) staining was performed. The sciatic nerves were examined under a light microscope. The ImageJ program (Bethesda, MD, USA) was used to measure the thickness of axons and calculate their number in a 2,500 µm^2^ area. For assessing the intact structure, the myelin in the sciatic nerve LFB stain was observed under a light microscope.

Quantitative polymerase chain reaction (qPCR)

Real-time polymerase chain reaction (RT-PCR) was used to analyze the gene expression of Nrf2 and β-actin (housekeeping gene). On day 28, the animals were sacrificed with an overdose of an anesthetic agent, and sciatic nerves were harvested. The total RNA was isolated with TRIzol (Invitrogen, USA) as per the manufacturer’s instructions. The quality and quantity of the total RNA isolated were assured by NanoQuant Plate™ using Tecan Infinite F200 Pro Microplate Reader (Tecan, Switzerland). GoTaq® 2-Step RT-qPCR System (Promega, USA) was used for gene expression. cDNA was synthesized from the isolated mRNA using GoScript™ Reverse Transcriptase. RT-PCR (quantitative) was carried out using the GoTaq® qPCR Master Mix (Promega, USA) in Rotor-Gene Q (Qiagen, Germany) machine. The cycle threshold (CT) value of each sample was noted. Fold change in different groups was calculated using the 2−ΔΔCT method [19].

Statistical analysis

The data obtained are expressed as mean ± standard error of the mean (SEM). Two-way analysis of variance (ANOVA) followed by a post hoc Tukey test was used to test the difference at different time points within the group in the case of behavioral studies such as hot plate, tail immersion, and von Frey tests. One-way ANOVA followed by a post hoc Tukey test was used to statistically compare pro-inflammatory cytokines, p38 MAPK, BDNF, axon thickness, number of axons, and mRNA levels between the experimental groups. All tests were performed using GraphPad Prism 8.4.3.686 software (San Diego, California, USA).

## Results

Effect of dimethyl fumarate (DMF) on thermal hyperalgesia

The PTX treatment significantly decreased the escape latency from Eddy’s hot plate on day 7 (7.77 ± 0.76), day 14 (7.44 ± 0.26), day 21 (7.50 ± 0.41), and day 28 (7.77 ± 0.31) compared to baseline latency (15.38 ± 0.92) (Table 2), which indicates that PTX-administered animals developed thermal hyperalgesia. In contrast, the coadministration of DMF 25 mg/kg and DMF 50 mg/kg significantly attenuated PTX-induced thermal hyperalgesia, which indicates the protective effect of DMF. The escape latency did not decline significantly from day 7 (13.33 ± 0.58) to day 28 (14.83 ± 0.54) as compared to day 0 (16.11 ± 0.36) (Table 2). In GBP-treated animals, the improvement in latency time started from day 14 onward because a significant increase in escape latency was observed.

**Table 2:** Effect of dimethyl fumarate treatment on thermal hyperalgesia in paclitaxel-induced peripheral neuropathy DMF attenuated paclitaxel-induced thermal hyperalgesia. To determine the significance of the statistical difference in escape latency at various testing time intervals within each experimental group and between the groups, a two-way ANOVA test was employed, followed by a Tukey post hoc test. *p < 0.05 and **p < 0.001 indicate the significance of the comparison between baseline reading (day 0) and post-paclitaxel administered readings. ^##^p < 0.0001 indicates the statistical significance in the vehicle control and the PTX + CMC 0.5% groups. ^†^p < 0.001 and ^††^p < 0.0001 indicate the statistical difference between PTX + CMC 0.5% and the respective treatment groups. Values are latency time in seconds. DMF: dimethyl fumarate; PTX: paclitaxel; CMC: carboxymethyl cellulose; ANOVA: analysis of variance

	Vehicle control (CMC 0.5%)	PTX (2 mg/kg) + CMC 0.5%	PTX + gabapentin 75 mg/kg	PTX + dimethyl fumarate 25 mg/kg	PTX + dimethyl fumarate 50 mg/kg
Day 0	15.16 ± 1.13	15.38 ± 0.92	16.11 ± 0.36	16.11 ± 0.36	14.22 ± 0.93
Day 7	12.10 ± 0.70*	07.77 ± 0.76**^##^	10.83 ± 0.41**†	13.33 ± 0.58^††^	13.66 ± 1.7^††^
Day 14	13.83 ± 0.54	07.44 ± 0.26**^##^	13.11 ± 0.32*^††^	13.83 ± 0.76^††^	15.11 ± 1.13^††^
Day 21	13.83 ± 0.30	07.50 ± 0.41**^##^	14.55 ± 0.34^††^	14.67 ± 0.76^††^	16.22 ± 1.03^††^
Day 28	12.84 ± 0.30	07.77 ± 0.31**^##^	14.55 ± 0.34^††^	14.83 ± 0.54^††^	15.00 ± 0.60^††^

Effect of DMF on tail cold allodynia

Table 3 represents the effect of DMF and GBP on cold allodynia. Animals administered with PTX experienced a reduction in the latency on day 14 (7.66 ± 0.66) and day 28 (8.66 ± 0.88) in comparison to day 0, which was statistically significant (Table 3). This decline in the latency time indicates that PTX-treated animals developed cold allodynia. However, the coadministration of GBP (75 mg/kg), DMF (25 mg/kg), and DMF (50 mg/kg) significantly improved PTX-induced cold allodynia, and this implies protection by GBP and DMF.

**Table 3:** Effect of dimethyl fumarate on cold allodynia in paclitaxel-induced peripheral neuropathy DMF treatment attenuated paclitaxel-induced cold allodynia. To determine the significance of the statistical difference in tail-flick latency to the cold sensitivity at various testing time points within each experimental group, a two-way ANOVA was performed, followed by a Tukey post hoc test. *p < 0.01 and **p < 0.0001 indicate the significance of the comparison between baseline reading (day 0) and post-paclitaxel administered readings. ^#^p < 0.05 indicates the statistical significance in the vehicle control and the PTX + CMC 0.5% groups. ^†^p < 0.05 indicates the statistical difference between PTX + CMC 0.5% and the respective treatment groups. Values are latency time in seconds. DMF: dimethyl fumarate; PTX: paclitaxel; CMC: carboxymethyl cellulose; ANOVA: analysis of variance

	Vehicle control (CMC 0.5%)	PTX (2 mg/kg) + CMC 0.5%	PTX + gabapentin 75 mg/kg	PTX + dimethyl fumarate 25 mg/kg	PTX + dimethyl fumarate 50 mg/kg
Day 0	12.50 ± 0.80	12.83 ± 0.60	11.66 ± 0.66	13.16 ± 0.87	12.00 ± 0.77
Day 7	11.50 ± 0.56	09.50 ± 0.99	11.66 ± 0.71	11.16 ± 0.83	10.66 ± 0.76
Day 14	12.66 ± 0.33	07.66 ± 0.66**^#^	08.50 ± 1.23	10.16 ± 0.87	11.50 ± 0.61^†^
Day 21	13.16 ± 1.35	09.33 ± 0.80^#^	09.83 ± 1.10	11.66 ± 1.28	13.33 ± 1.08^†^
Day 28	12.83 ± 0.83	08.66 ± 0.88*^#^	11.16 ± 1.13	11.83 ± 1.24	13.83 ± 0.83^†^

Effect of DMF on mechanical allodynia

Paclitaxel (2 mg/kg) significantly shortened the paw withdrawal threshold on day 7 (20.5 ± 2.45 g), day 21 (18.66 ± 2.31 g), and day 28 (15.16 ± 0.16 g) as compared to the baseline readings on day 0 (43 ± 7.6 g). Nonetheless, GBP and DMF significantly delayed the paw withdrawal when coadministered with PTX (Table 4).

**Table 4:** Effect of dimethyl fumarate on mechanical allodynia in paclitaxel-induced peripheral neuropathy Data are expressed as mean ± SEM. To determine the significance of the statistical difference in paw withdrawal threshold to the mechanical stimulation at various testing time points within each experimental group, two-way ANOVA was employed, followed by a Tukey post hoc test. *p < 0.05 and **p < 0.001 indicate the significance of the comparison between baseline reading (day 0) and post-paclitaxel administered readings. ^#^p < 0.05 and ^##^p < 0.005 indicate the statistical significance in the vehicle control and the PTX + CMC 0.5% groups. ^†^p < 0.005 and ^††^p < 0.0005 indicate the statistical difference between PTX + CMC 0.5% and the respective treatment groups. Values are latency time in seconds. DMF: dimethyl fumarate; PTX: paclitaxel; CMC: carboxymethyl cellulose; ANOVA: analysis of variance; SEM: standard error of the mean

	Vehicle control (CMC 0.5%)	PTX (2 mg/kg) + CMC 0.5%	PTX + gabapentin 75 mg/kg	PTX + dimethyl fumarate 25 mg/kg	PTX + dimethyl fumarate 50 mg/kg
Day 0	52.50 ± 7.50	43.00 ± 7.60	37.33 ± 7.16	66.66 ± 6.66	60.00 ± 00.00
Day 7	54.33 ± 5.66	20.50 ± 2.45*^##^	41.16 ± 8.58	41.16 ± 8.58	53.50 ± 12.28^†^
Day 14	48.66 ± 7.16	20.50 ± 2.45*^#^	43.00 ± 7.60	54.33 ± 5.66^†^	60.00 ± 00.00^†^
Day 21	61.00 ± 9.57	18.66 ± 2.31*^##^	48.66 ± 7.16^†^	54.33 ± 5.66^†^	54.33 ± 05.66^†^
Day 28	48.66 ± 7.16	15.16 ± 0.16*^##^	48.66 ± 7.16^†^	54.33 ± 5.66^†^	54.33 ± 05.66^†^

Effect of DMF on pro-inflammatory cytokines

**Figure 1:**
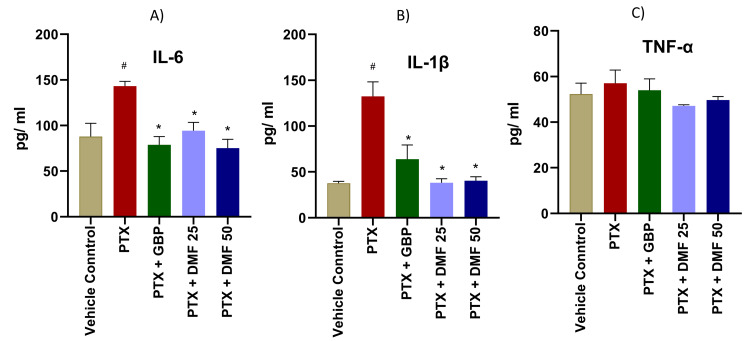
Effect of DMF treatment on IL-6, IL-1β, and TNF-α in PTX-induced peripheral neuropathy rat model IL-6 (A), IL-1β (B), and TNF-α (C) concentrations in different groups were measured using the ELISA method. Data are expressed as mean ± SEM. One-way ANOVA followed by Tukey post hoc analysis was done. # indicates significance (p < 0.05) in between CMC (vehicle) and PTX + CMC group; * indicates significance (p < 0.05) with the PTX + CMC 0.5% group. IL: interleukin; TNF-α: tumor necrosis factor-alpha; PTX: paclitaxel; GBP: gabapentin; DMF: dimethyl fumarate; ELISA: enzyme-linked immunosorbent assay; SEM: standard error of the mean; ANOVA: analysis of variance; CMC: carboxymethyl cellulose

The results revealed that IL-6 and IL-1β were significantly elevated in the paclitaxel group. DMF and GBP significantly blunted the effect of PTX on the levels of PTX IL-6 and IL-1β (Figure 1). Nonetheless, TNF-α did not significantly change in PTX-treated animals as compared to the control group (Figure 1).

Effect of DMF on BDNF and p38 MAPK

**Figure 2:**
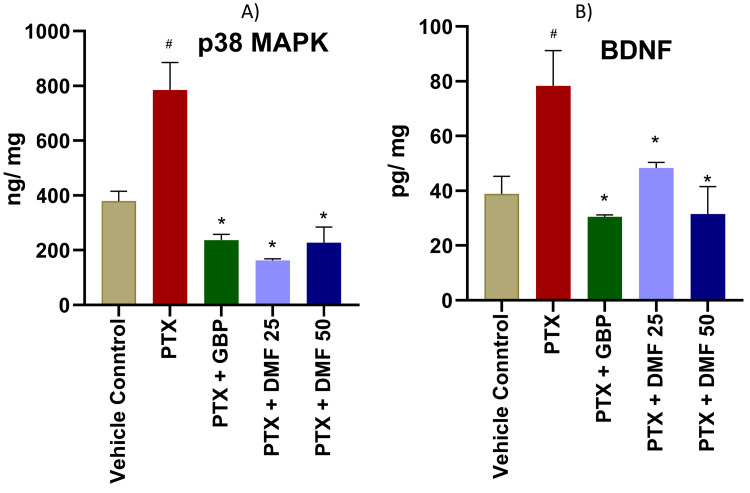
Effect of DMF treatment on p38 MAPK and BDNF in the dorsal horn of spinal cord of rats with PTX-induced peripheral neuropathy p38 MAPK (A) and BDNF (B) concentrations in different groups were measured using the ELISA method. Data are expressed as mean ± SEM. One-way ANOVA followed by Tukey post hoc analysis was done. # indicates significance (p < 0.05) between CMC (vehicle) and PTX + CMC group; * indicates significance (p < 0.05) with the PTX + CMC 0.5% group. DMF: dimethyl fumarate; PTX: paclitaxel; GBP: gabapentin; MAPK: mitogen-activated protein kinase; BDNF: brain-derived neurotrophic factor; ELISA: enzyme-linked immunosorbent assay; SEM: standard error of the mean; ANOVA: analysis of variance; CMC: carboxymethyl cellulose

The levels of p38 MAPK and BDNF in the dorsal horn of the spinal cord are presented in Figure 2. The study revealed that paclitaxel treatment increased the expression of p38 MAPK and BDNF compared to the control group. However, DMF and GBP attenuated the paclitaxel-induced increase in p38 MAPK and BDNF levels in the spinal cord’s dorsal horns.

Effect of DMF on the gene expression of Nrf2/β-actin

**Figure 3:**
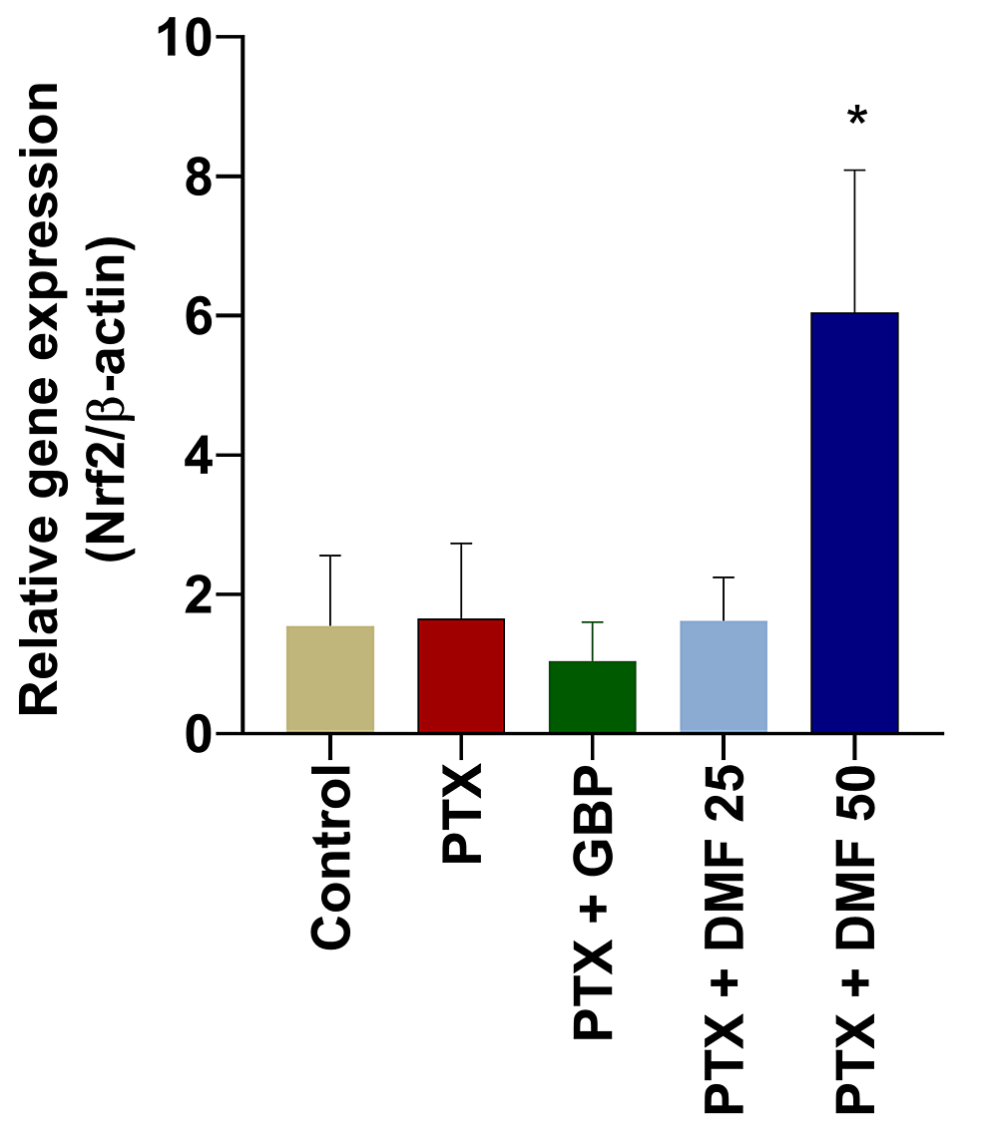
Effect of DMF treatment on relative gene expression (Nrf2/β-actin) in the sciatic nerves of the rats with paclitaxel-induced peripheral neuropathy Nrf2 and β-actin gene expressions were analyzed by RT-PCR. Data are expressed as mean ± SEM. One-way ANOVA followed by Tukey post hoc analysis was done. * indicates significance (p < 0.05) with the PTX + CMC 0.5% group. Nrf2: nuclear factor erythroid-2-related factor 2; PTX: paclitaxel; GBP: gabapentin; DMF: dimethyl fumarate; RT-PCR: real-time polymerase chain reaction; SEM: standard error of the mean; ANOVA: analysis of variance; CMC: carboxymethyl cellulose

The relative gene expression of Nrf2 in the sciatic nerve of rats of various groups is shown in Figure 3. PTX did not significantly change the expression of Nrf2 as compared to the vehicle control group. Also, no significant change in the GBP + PTX group was seen. However, DMF 50 mg/kg treatment significantly increased the expression of Nrf2 in the paclitaxel-treated rats.

Effect of DMF on histopathological changes

**Figure 4:**
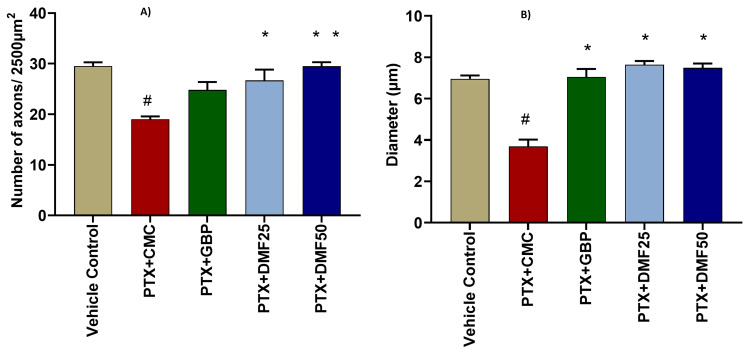
Effect of DMF treatment on the number of axons/2 500 µm2 and axon diameter (μm) in paclitaxel-induced peripheral neuropathy rat model The number of axons/2500 µm^2^ (A) and axon diameter (μm) (B) in different groups were measured using H&E staining of rat sciatic nerve. Data are expressed in mean ± SEM. # indicates significance (p < 0.05) in between CMC (vehicle) and PTX + CMC group; * indicates significance (p < 0.0001) with the PTX + CMC 0.5% group. PTX: paclitaxel; GBP: gabapentin; DMF: dimethyl fumarate; CMC: carboxymethyl cellulose; H&E: hematoxylin and eosin; SEM: standard error of the mean

**Figure 5:**
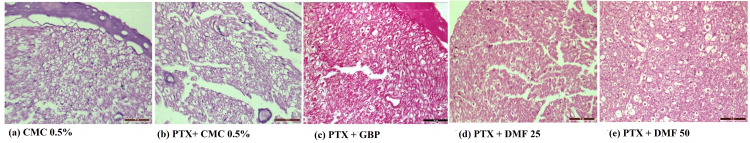
Photomicrograph (40×) H&E stain of rat sciatic nerve (transverse section) showing neural morphology in different experimental groups Vehicle (CMC 0.5%) (a) group showing normal sciatic nerve morphology. PTX + CMC 0.5% (b) group showing swollen axons and myelin damage. PTX + GBP (c) group showing near to normal nerve morphology. PTX + DMF 25 mg/kg (d) group showing less swollen axons. PTX + DMF 50 mg/kg (e) group showing less swollen axons as compared to PTX + CMC 0.5% group. CMC: carboxymethyl cellulose; PTX: paclitaxel; GBP: gabapentin; DMF: dimethyl fumarate; H&E: hematoxylin and eosin

The axonal diameter in the animals treated with PTX 2 mg/kg + CMC 0.5% was reduced, and that reduction was statistically significant in comparison to the vehicle control group (Figure 4 and Figure 5). On the other hand, a statistically significant increase in the diameter was observed in the PTX 2 mg/kg + GBP 5 mg/kg treatment group in comparison to the PTX 2 mg/kg + CMC 0.5% treatment group. Likewise, in paclitaxel 2 mg/kg + dimethyl fumarate 25 mg/kg animals, the axonal diameter was comparatively high as compared to the animals treated with PTX 2 mg/kg + CMC 0.5%. Also, in paclitaxel 2 mg/kg + dimethyl fumarate 50 mg/kg animals, the axonal diameter was greater than in the group treated with PTX 2 mg/kg + CMC 0.5%. The change in both the DMF-treated groups was statistically significant.

## Discussion

Chemotherapy-induced peripheral neuropathy is an utmost side effect encountered through several drugs used to treat cancer. Although each drug has a different mechanism of action in cancer, they all produce oxidative stress, the main contributor to peripheral neuropathy. Likewise, the microtubule stabilizer paclitaxel results in peripheral neuropathy (PIPN). Currently, 7%-10% of the global population suffers from peripheral neuropathy [2,3,22].

In this study, paclitaxel treatment (2 mg/kg injection on days 0, 2, 4, and 6) caused neuropathy manifesting in the form of thermal hyperalgesia, mechanical allodynia, and tail cold allodynia. The same has already been reported in previous studies with different doses of paclitaxel in different animal models [1,7,23-25]. GBP prevented the development of PTX-induced thermal hyperalgesia, mechanical allodynia, and tail cold allodynia in the rats. The protective effects of GBP in PTX-induced neuropathy have already been reported in clinical and nonclinical studies [26-28]. DMF (25 mg/kg and 50 mg/kg) effectively ameliorated the thermal hyperalgesia, mechanical allodynia, and tail cold allodynia induced by PTX. Likewise, past studies observed a similar effect of DMF in other models of chemotherapy-induced peripheral neuropathy [29-31].

Recent research has shown central sensitization’s role in worsening neuropathic pain [8,32]. Microglial cells in the spinal cord dorsal horn play a substantial role in the induction of central sensitization [33]. BDNF is a neuropeptide that has been reported to exacerbate central sensitization [34-36]. The release of BDNF increases from microglial cells in the dorsal horn of the spinal cord whenever there is a peripheral nerve injury mediated by the increased expression of the ATP-gated ionotropic purinoceptor 2X4 (P2X4R); this increase in expression parallels the increase in pain hypersensitivity [2]. The BDNF released from microglia is dependent on the influx of Ca^2+^via P2X4Rs, which activates p38 MAPK, resulting in the increased production and release of BDNF [13].

As reported by previous mechanistic studies to link BDNF and p38 MAPK, BDNF and p38 MAPK levels in the spinal cord dorsal horn of neuropathic rats in the paclitaxel group were significantly high as compared to the control group [12,37,38]. Nevertheless, DMF treatment significantly attenuated the effect of paclitaxel on p38 MAPK and BDNF. Decreased BDNF levels could be the effect of p38 MAPK inhibition by DMF, which is already known [17,39]. The inhibition of the phosphorylation of p38 MAPK can further inhibit the production and exocytosis of BDNF in the spinal cord dorsal horn [12].

IL-1β, IL-6, and TNF-α pro-inflammatory cytokines are factors responsible for neuroinflammation in peripheral and central neuropathy. Other than axonal damage, pro-inflammatory cytokines change spontaneous nociception and stimulus sensitivity. In the sensory neurons, p38 MAPK and Jun-N-terminal kinase (JNK) phosphorylation is initiated by the activation of TNF receptors and the hiring of TNF receptor-associated factors (TRAFs), which possibly activates nuclear factor-kappa B (NF-kB) [40].

Paclitaxel and other chemotherapeutic agents cause many histopathological changes in the peripheral nerves. Therefore, we examined the histopathology of the sciatic nerve in peripheral neuropathy and the effect of DMF treatments. The PTX treatment significantly reduced the number of axons and the axonal diameter in the sciatic nerve. A recent study has also reported the same in PINP [7]. The gabapentin treatment group, compared to the PTX group, significantly retained the axonal diameter and the number of axons per 2,500-micrometer area. Likewise, DMF also preserved the number of axons and the axonal diameter in the DMF-treated groups. Our results were similar to the previous studies, where GBP and DMF have been reported to be neuroprotective [41,42].

This is an established fact that DMF acts by the activation of Nrf2; as a result, it increases the expression of antioxidant proteins. Nrf2 expression in the PTX- and GBP-treated group was not changed. In the DMF 50 mg/kg treatment group, however, a significant increase in the Nrf2 expression was seen. These findings were parallel to the previously reported evidence of DMF in neurological disorders [15,16,43].

In the neuropathic rats treated with paclitaxel in this study, IL-1β and IL-6 levels significantly increased. On the other hand, significant protection against the paclitaxel-induced rise in IL-1β and IL-6 was demonstrated by DMF and GBP. This effect could be because of the p38 MAPK activation inhibition property of DMF and GBP, which is a crucial step in the production of intracellular pro-inflammatory cytokines [17,44-46].

It is a known fact that DMF shows neuroprotection by activating the Nrf2 cascade of antioxidant proteins. In the Nrf2 knockout mouse, the DMF showed minimal or no effect [15,16,43]. In the present study, we examined whether paclitaxel-induced peripheral neuropathy is ameliorated by activating the Nrf2 gene in rats administered with DMF.

## Conclusions

The study demonstrated that DMF shows significant protection against paclitaxel-induced peripheral neuropathy in rats. The reduction in pro-inflammatory cytokines and increases in the Nrf2 gene expression suggest that DMF curbed inflammation and increased antioxidant defense. Furthermore, the reduced p38 MAPK and BDNF in the dorsal horn of the spinal cord indicate that DMF might be acting by decreasing the central sensitization of neuropathic pain.
